# A Commitment to HIV Diagnostic Accuracy – a comment on “Towards more accurate HIV testing in sub‐Saharan Africa: a multi‐site evaluation of HIV RDTs and risk factors for false positives ‘and’ HIV misdiagnosis in sub‐Saharan Africa: a performance of diagnostic algorithms at six testing sites”

**DOI:** 10.1002/jia2.25177

**Published:** 2018-08-31

**Authors:** Amy S Kravitz Del Solar, Bharat Parekh, Meaghan O'Keefe Douglas, Dianna Edgil, Joel Kuritsky, John Nkengasong

**Affiliations:** ^1^ Office of HIV/AIDS United States Agency for International Development Washington DC USA; ^2^ Division of Global HIV and TB Centers for Disease Control and Prevention Atlanta GA USA; ^3^ Africa Centers for Disease Control and Prevention Africa Union Addis Ababa Ethiopia

## Abstract

As part of the global response to the HIV/AIDS epidemic, the U.S. President's Emergency Plan for AIDS Relief (PEPFAR) is committed to the provision of high‐quality services and ensuring testing accuracy. Two recently published papers focusing on HIV testing and misdiagnosis in sub‐Saharan Africa by Kosack *et al*. report on evaluations of HIV rapid diagnostic tests (RDTs) and found lower than expected specificity and sensitivity on some tests when used in certain geographic locations. The magnitude of PEPFAR's global HIV response has been possible due to the extensive use of RDTs, which have made HIV diagnosis accessible all over the world. We take the opportunity to address concerns raised about the potential implications that these findings could have on real‐world HIV testing accuracy. PEPFAR supported countries adhere to the normative guidance by World Health Organization (WHO) supporting algorithms which require sequential positive tests for diagnostic accuracy. An analysis of Médecins Sans Frontières (MSF) RDT site‐specific data applied to PEPFAR in‐country protocols demonstrate a variation in the diagnostic accuracy of the testing algorithms, but with a very small population‐level effect. The data demonstrate, with the use of these algorithms, that the RDT outcomes found in the study by Kosack *et al*. would be largely mitigated and would not be expected to have a significant impact on diagnostic accuracy and overall programming in most countries. Avoiding any misdiagnosis is a priority for PEPFAR, and it remains vital to gain a deeper understanding of the causes and the extent of diagnostic errors and any misclassification. Extensive quality control mechanisms and continued research are essential. With a focus on epidemic control and ensuring diagnostic accuracy, PEPFAR recommends that all countries use WHO pre‐qualified RDTs within the recommended strategies and algorithms for HIV testing. We also support validation of HIV testing algorithms using in‐country specimens to determine optimal performance, and the reverification testing of all people diagnosed with HIV prior to starting treatment as an essential quality assurance measure.

## 1 Introduction

The recently published papers, *Towards more accurate HIV testing in sub‐Saharan Africa: a multi‐site evaluation of HIV RDTs and risk factors for false positives* and *HIV misdiagnosis in sub‐Saharan Africa: performance of diagnostic algorithms at six testing sites* by Kosack *et al*. describe evaluations of several HIV rapid diagnostic tests (RDTs) and confirmatory assays both alone and in series. The authors report findings of lower than expected sensitivity and specificity on some tests when used in certain geographical locations 1, 2. While the tests evaluated in this study had all previously passed World Health Organization (WHO) performance criteria (≥99% sensitivity and ≥98% specificity), the evaluation showed that the individual “RDTs performed more poorly than in the WHO evaluations.” With questions being raised about the potential impact of these results in real‐world HIV testing services, we would like to take the opportunity to address this issue and its potential relevance with our HIV testing in U.S. President's Emergency Plan for AIDS Relief (PEPFAR) programmes.

Since its inception in 2003, PEPFAR has shown an unwavering commitment in the response for the global HIV/AIDS crisis, working in nearly 60 countries. As of September 2017, PEPFAR has provided testing services to 85.5 million people, and 13.3 million HIV‐positive men, women and children are supported on life‐saving antiretroviral treatment 3. The expanding success of this programme has been achieved by using data to drive accountability, and we welcome the continued development of information to inform our programming and further the global HIV response.

The magnitude of this scale‐up has been made possible through the use of RDTs, some of which were examined by the Kosack *et al*. evaluations. These HIV serology assays detect the presence of HIV‐1/2 antibodies and have had high sensitivity and specificity compared with assays for other infectious diseases 4. RDTs have been instrumental for increased access to HIV testing, allowing testing to be performed in both community‐ and facility‐based settings, including sites with limited infrastructure that process low numbers of specimens daily. Critically, the relatively easy use and transportability of RDTs has resulted in higher rates of diagnoses globally, through more patients tested. Moreover, the availability of RTKs has assisted in moving the proportion of those who know their HIV positivity status from an estimated 10% in 2004 in Sub‐Saharan Africa to 76% as of 2016 in East and Southern Africa 5, 6. Many countries are approaching 90% of people living with HIV (PLHIV) knowing their status, a key Joint United Nations Programme on HIV/AIDS (UNAIDS) benchmark 7.

## 2 Discussion

It has been well established, however, that a single reactive HIV test is not sufficient to provide an HIV‐positive diagnosis. Irrespective of assay format, false‐reactive test results can occur due to user error, manufacturing errors (i.e. lapses in quality management systems), or biological factors (e.g. cross‐reactive antibodies, contaminating proteins). To provide a definitive HIV‐positive diagnosis, normative guidance by WHO emphasizes the importance of using one of two different testing strategies according to the HIV prevalence in the setting to provide an HIV diagnosis:

- In a high‐prevalence (≥5%) setting, two sequential different reactive (positive) tests are needed to provide a person with an HIV‐positive diagnosis.
- In a low‐prevalence (<5%) setting, three sequential different reactive (positive) tests are needed to provide a person with an HIV‐positive diagnosis.

Use of these algorithms has been shown to provide reliably accurate diagnosis, comparable to ELISA followed by Immunoblot (Western blot) 8. The cited studies by Kosack *et al*., however, show unusual variation in the performance of RDTs used individually, and in some cases, in series by populations and settings. For Kosack *et al*., the use of RDTs in series and according to the national algorithms largely eliminated incorrect diagnosis, with certain exceptions corresponding to location and tests used. In the light of these results, and given that most PEPFAR‐supported countries adhere to this algorithmic approach, the frequency of false‐positive diagnoses would be mitigated, but not eliminated entirely. The extent to which this might be an issue is calculated below.

### 2.1 Results analysis

Validation of the testing‐specific algorithms used in each individual setting requires parallel testing of a status quo testing algorithm in comparison with a candidate testing algorithm to determine rate of misdiagnosis, and so the data obtained by Kosack *et al*. cannot be accurately applied to the broader context. As PEPFAR works in five of the six sites analysed by Kosack *et al*., and has details about the site‐specific performance information, including algorithm usage and prevalence data, our analysis at those five sites demonstrates the practical impact of the individual RTK results in a real‐world setting. Analysis of the data available through the Kosack *et al*. publications, applied to PEPFAR data on prevalence and national testing algorithms, has been used to demonstrate the practical implications and impact of those findings on testing programmes. Exploratory modelling examining the Médecins Sans Frontières (MSF) RDT site‐specific data presented in their published papers, applied to the in‐country protocols do show some variation in diagnostic accuracy of algorithms, but this has only a very small population‐level effect (Tables 1 and 2). Even using the sensitivity and specificity data from the lower end of the confidence interval found in the MSF study, one potential false‐positive result would necessitate performing thousands of tests, with some sites requiring testing in the tens of thousands. This figure would be even further reduced with the utilization of verification testing before antiretroviral therapy (ART), as is currently recommended by WHO and in all PEPFAR‐supported countries. Moreover, it is significant to note that when using data from the lower end of the confidence interval, there is a potential decrease in diagnostic accuracy of the algorithms. However, if the data from the upper end of the confidence interval are used, the algorithms often outperform what could be anticipated, even in comparison to the manufacturers’ performance claims stated in the instructions for use. These wide confidence intervals found in this analysis, likely attributed to the sample size, make definitive conclusions impossible to draw, but do underline the need improved validation in this area. Based on the analysis of the available data, the RDT outcomes from the study would likely have a small to negligible impact on our case finding results, diagnostic accuracy and overall programming in most countries.

**Table 1:** Kosack *et al*. data calculated to illustrate the algorithms’ positive predictive value^a^

Site	HIV prevalence	Algorithm	Sensitivity				Algorithm PPV		
			Lowest bound of performance based on low confidence interval (MSF data)	Highest bound of performance based on high confidence interval (MSF data)	Point estimate of confidence interval (MSF data)	Manufacturer data	Worst case	Best case	Point estimate of confidence interval (MSF data)
Guinea, Conakry	2.7%	Determine	98.30%	100%	100%	99.9%	98.89%	99.98%	99.89%
		SD Bioline	98.30%	100%	100%	100%			
Uganda, Kitgum	8.3%	Determine	98.3%	100%	100%	99.9%	97.3%	100%	100.0%
		HIV STAT‐PAK	77.9%	99.5%	96.2%	99.7%			
		Uni‐Gold	77.9%	99.5%	96.2%	100%			
Uganda, Arua	4.9%	Determine	98.3%	100%	100%	99.9%	99.1%	100.0%	99.9%
		HIV STAT‐PAK	98.3%	100%	100%	99.7%			
		Uni‐Gold	98.3%	100%	100%	100%			
Kenya, Homa Bay	26%	Determine	98.3%	100%	100%	99.9%	95.1%	98.9%	97.7%
		First Response	98.3%	100%	100%	99.4%			
		Uni‐Gold	96.8%	99.9%	99.6%	100%			
DRC, Baraka	0.8%	Determine	98.3%	100%	100%	99.9%	93.6%	99.8%	98.9%
		Uni‐Gold	96.8%	99.9%	99.6%	100%			
		Vikia	96.8%	99.9%	99.6%	99.95%			

a Estimates for the algorithm assume that test results at each step are independent of those in the prior step; worst case and best case performance estimates were calculated using the lower and upper 95% bounds for each test respectively.

**Table 2:** Kosack *et al*. data calculated to illustrate the algorithms’ negative predictive value^a^

Site	HIV prevalence	Algorithm	Specificity				Algorithm NPV		
			Lowest end of confidence interval (MSF data)	Highest end of confidence interval (MSF data)	Point estimate of confidence interval (MSF data)	Manufacturer data	Worst case	Best case	Point estimate of confidence interval (MSF data)
Guinea, Conakry	2.7%	Determine	97.70%	99.6%	99%	98.2%	99.9%	100%	100%
		SD Bioline	98.70%	99.9%	99.7%	99.8%			
Uganda, Kitgum	8.3%	Determine	88.8%	95.8%	93.1%	98.2%	99.4%	100%	100.0%
		HIV STAT‐PAK	98.3%	100.0%	100.0%	99.9%			
		Uni‐Gold	95.2%	99.3%	98.2%	100%			
Uganda, Arua	4.9%	Determine	90.6%	96.8%	94.4%	98.2%	99.9%	100.0%	100.0%
		HIV STAT‐PAK	99.5%	100.0%	99.9%	99.9%			
		Uni‐Gold	93.7%	98.5%	96.9%	100%			
Kenya, Homa Bay	26%	Determine	91.0%	96.5%	94.4%	98.2%	99.4%	100.0%	100.0%
		First Response	80.6%	89.0%	85.3%	99.4%			
		Uni‐Gold	96.9%	99.7%	99.0%	99.8%			
DRC, Baraka	0.8%	Determine	87.8%	94.7%	91.9%	98.2%	100.0%	100.0%	100.0%
		Uni‐Gold	93.3%	98.2%	96.5%	100%			
		Vikia	93.8%	98.4%	96.8%	99.86%			

a Estimates for the algorithm assume that test results at each step are independent of those in the prior step; worst case and best case performance estimates were calculated using the lower and upper 95% bounds for each test respectively.

The results of this study could provide new insight into those factors which affect testing accuracy on particular individual RDTs – specifically, the possibility of a stronger influence by geographical and population differences on serology assay performance. This supports previous work that suggests that biological factors within the population (i.e. cross‐reactivity of antigens, non‐specific IgG binding or contaminating proteins in specimens), may play a more prominent role in the performance of some RDTs than initially thought 9. This is something of interest for the refinement of algorithms for programmatic implementation based on the epidemiological profile of the population, and eventually, individual characteristics, as well as product research and development.

### 2.2 Misdiagnosis rates

The consequences of any misdiagnoses of HIV status are serious, with negative impacts on both the individual and the health system. Ensuring the accuracy of the HIV‐testing process has been a priority for the global community and extensive quality assurance mechanisms have been put in place. From a PEPFAR perspective, these include lot verification testing as a form of post‐market surveillance of all U.S. Agency for International Development (USAID)‐procured RDTs prior to shipment, USG‐conducted Site Improvement through Monitoring System (SIMS) supervision visits, which use a standardized tool to annually assess programme quality across the HIV portfolio including implementation of regular HIV proficiency testing to verify user performance at all PEPFAR high‐volume testing sites 10. Annually, the USG conducts tens of thousands of SIMS assessments in PEPFAR countries to ensure programme quality. Furthermore, a number of additional tools have been developed and implemented to monitor and ensure accuracy of HIV rapid testing with focus on user training and proficiency 11.

To increase algorithm effectiveness, gaining an understanding of patient comorbidities that result in false‐reactive HIV test results is critical. HIV cross‐reactive antibodies have been reported for several conditions, including TB, malaria, leprosy and rheumatoid arthritis 12. While the authors do mention the potential influence of unidentified demographic factors, additional research is warranted into the prevalence and aetiology of false reactivity in key populations or those in certain geographical locations as well as programmatic validation of testing algorithms to decrease the potential of false reactivity. As well, the selection and validation of country algorithms should take into account and seek to eliminate the potential overlap in antigen sourcing among different manufacturers of RDTs. The use of a specimen panel containing identified falsely reactive specimens is warranted but difficult to obtain. The global community should consider the establishment of a global panel of characterized falsely reactive specimens for use in country validation.

### 2.3 Potential quality issues affecting HIV rapid test results

The results by Kosack *et al*. provide important new clues in the understanding of diagnostic accuracy, as previous reports of misdiagnosis in resource‐limited settings have been primarily hypothesized to be the result of factors such as user errors 13. Some of these are, in theory, preventable (improper adherence to the instructions for use issued by the assay manufacturer, improper specimen handling, clerical errors, etc.) and would be minimized by better training and through verification retesting of any individual diagnosed as HIV positive prior to ART initiation. Suboptimal testing strategies (lack of adherence to algorithms or use of incorrect algorithms such as use of result of Assay 3 as a “tie breaker” test to rule in infection) have also been attributed to inaccurate diagnosis reinforcing the importance of adherence to recommended testing strategies 9. For many countries, algorithm validation using local clinical specimens is not done in series and does not include an assessment of overlap in RDT specificity that might decrease the probability Assay 1 and Assay 2 (or Assay 3) falsely reacting with a patient specimen 14.

Of note, while any visually read assay is prone to inter‐reader variability, RDTs also have specifications in terms of storage temperature, assay robustness (including volume of specimen and buffer used, mixing techniques, etc.) and incubation times. Rigorous quality control must be in place to ensure these factors are controlled and minimized. Kosack *et al*. used plasma samples that had been frozen, shipped and stored, whereas RDTs, while often validated for serum and plasma, are designed to be used with fresh specimens, typically capillary whole blood. While the limited data available do not indicate clear discordance with the testing results from previously frozen samples, as noted by the authors, “some studies have shown differences in sensitivity and specificity when using plasma/serum compared to capillary whole blood,” which could be postulated to have had an impact on the results seen 15, 16, 17.

## 3 Conclusions

As we aim for epidemic control and meeting UNAIDS’ 90‐90‐90 targets, the three pillars of PEPFAR programmes are accountability, transparency and impact. With these priorities in mind, we publically share all available levels of programme data with the ultimate aim of saving lives and averting new infections. We welcome the continued development of data which could enhance the effectiveness of HIV programming.

Considering the currently available evidence, we recommend that all countries continue to use WHO‐prequalified RDTs within the recommended testing strategies, and support member states validating HIV‐testing algorithms using in‐country specimens to identify assays which, in series, will provide optimal performance. We also support verification retesting for all people with an HIV‐positive diagnosis prior to starting on ART, as a critical quality assurance step to further ensure those starting ART are indeed HIV positive. We are actively supporting this in all PEPFAR implementation countries.

## Competing interests

The authors declare that they have no competing interests.

## Authors’ contributions

BP, DE, JK and ASK developed the initial framework for the manuscript; ASK prepared the initial outline and draft; all authors reviewed and provided revisions to the draft; MOD performed the data analysis and data interpretation; ASK incorporated revisions and coordinated the approval from all authors prior to submission; ASK and BP facilitated office clearance. All authors have read and approved the final version.

## Funding

This publication was supported by the U.S. President's Emergency Plan for AIDS Relief through the U.S. Agency for International Development and the U.S. Centers for Disease Control and Prevention.

## Disclaimer

The findings and conclusions in this report are those of the authors and do not necessarily represent the official position of participating federal Agencies, including the U.S. Agency for International Development and the U.S. Centers for Disease Control and Prevention.
